# Aldolase B Knockdown Prevents High Glucose-Induced Methylglyoxal Overproduction and Cellular Dysfunction in Endothelial Cells

**DOI:** 10.1371/journal.pone.0041495

**Published:** 2012-07-24

**Authors:** Jianghai Liu, Timothy Chun-Ping Mak, Ali Banigesh, Kaushik Desai, Rui Wang, Lingyun Wu

**Affiliations:** 1 Department of Pharmacology, College of Medicine, University of Saskatchewan, Saskatoon, Saskatchewan, Canada; 2 Department of Biology, Lakehead University, Thunder Bay, Ontario, Canada; 3 Department of Health Sciences, Lakehead University and Thunder Bay Regional Research Institute, Thunder Bay, Ontario, Canada; University of Toronto, Canada

## Abstract

We used cultured endothelial cells as a model to examine whether up-regulation of aldolase B and enhanced methylglyoxal (MG) formation play an important role in high glucose-induced overproduction of advanced glycosylation endproducts (AGEs), oxidative stress and cellular dysfunction. High glucose (25 mM) incubation up-regulated mRNA levels of aldose reductase (an enzyme converting glucose to fructose) and aldolase B (a key enzyme that catalyzes MG formation from fructose) and enhanced MG formation in human umbilical vein endothelial cells (HUVECs) and HUVEC-derived EA. hy926 cells. High glucose-increased MG production in EA. hy926 cells was completely prevented by siRNA knockdown of aldolase B, but unaffected by siRNA knockdown of aldolase A, an enzyme responsible for MG formation during glycolysis. In addition, inhibition of cytochrome P450 2E1 or semicarbazide-sensitive amine oxidase which produces MG during the metabolism of lipid and proteins, respectively, did not alter MG production. Both high glucose (25 mM) and MG (30, 100 µM) increased the formation of N(ε)-carboxyethyl-lysine (CEL, a MG-induced AGE), oxidative stress (determined by the generation of oxidized DCF, H_2_O_2_, protein carbonyls and 8-oxo-dG), *O*-GlcNAc modification (product of the hexosamine pathway), membrane protein kinase C activity and nuclear translocation of NF-κB in EA. hy926 cells. However, the above metabolic and signaling alterations induced by high glucose were completely prevented by knockdown of aldolase B and partially by application of aminoguanidine (a MG scavenger) or alagebrium (an AGEs breaker). In conclusion, efficient inhibition of aldolase B can prevent high glucose-induced overproduction of MG and related cellular dysfunction in endothelial cells.

## Introduction

Hyperglycemia in diabetes mellitus damages blood vessels and induces vascular complications in the retinal, renal, and cardiovascular tissues [1], [2]. Vascular endothelial cells are the early and primary targets of hyperglycemic damage in diabetes [3], [4]. Hyperglycemia-triggered endothelial dysfunction, including increased endothelial permeability and inflammation, decreased nitric oxide (NO) bioavailability and endothelium-dependent relaxation, and vascular remodeling, is considered a key event in the pathogenesis of diabetic vascular complications [5].

Methylglyoxal (MG) is a highly reactive metabolite of glucose [6], [7]. Increased MG levels were observed in vascular endothelial cells cultured in high glucose-containing media and in the aorta, kidney and retina of diabetic rats [8], [9], [10]. Accumulating evidence indicates that high glucose-increased MG production is an important molecular mechanism linking diabetes to endothelial damage. MG modifies lysine, arginine, and cysteine residues in peptides or proteins to yield irreversible advanced glycosylation end products (AGEs), leading to cross-linking and denaturation of proteins [11], [12], [13]. MG also increases the generation of reactive oxygen species (ROS) and oxidative stress in endothelial cells [8]. Moreover, indirect evidence implicates MG in the high glucose-activated protein kinase C (PKC), hexosamine, and nuclear factor κB (NF-κB) pathways. For example, AGEs activated PKC in cultured endothelial cells [14]; incubation with alagebrium, an AGEs breaker, reduced PKC activation in high glucose-treated vascular smooth muscle cells (VSMCs) [15]. Activation of the hexosamine pathway by hyperglycemia leads to O-linked N-acetyl glucosamine (*O*-GlcNAc) modification of various proteins on serine or threonine residues which impair the normal functions of proteins [1]. Overexpression of glyoxalase-1, an enzyme metabolizing MG, reduced high glucose-increased *O*-GlcNAc modification in endothelial cells [16]. NF-κB is activated by the elevation of glucose, leading to up-regulation of target genes relevant to endothelial inflammation and apoptosis [17], [18]. AGEs activated NF-κB in cultured endothelial cells [19]. A study in the cultured VSMCs showed that MG treatment induced the activation of NF-κB [20]. These data suggest that inhibition of MG production could be a strategy to prevent endothelial damage in diabetic vascular complications.

Several MG scavengers have been developed, but most of them, such as aminoguanidine, metformin and N-acetyl cysteine, are non-specific to MG and their utilization for scavenging MG and preventing diabetic damage is limited [21]. We recently identified aldolase B which converts glucose or fructose to MG as a primary enzyme responsible for MG overproduction in high glucose-treated VSMCs and in aorta of diabetic rats [6]. In this paper, the gene expression of aldolase B and its role in MG formation in high glucose-treated endothelial cells were evaluated and whether knockdown of aldolase B in endothelial cells prevented high glucose-induced MG overproduction and other metabolic and signaling abnormalities was investigated.

## Materials and Methods

### Cell Culture and Treatment

Human umbilical vein endothelial cells (HUVECs) from American Type Culture Collection were cultured in Kaighns F12K medium containing 10% fetal bovine serum (FBS), 0.1 mg/mL heparin and 0.03–0.05 mg/mL endothelial cell growth supplement. HUVECs between passage 3 and 6 were used for the experiments. EA. hy926 cells, a endothelial cell line derived from the fusion of HUVECs with A549 lung carcinoma cells [22] (a gift kindly provided by Dr. Cora-Jean Edgell, University of North Carolina at Chapel Hill), were cultured in Dulbecco’s modified Eagle’s medium (DMEM) supplemented with 10% FBS. EA. hy926 cells will retain endothelial phenotype and functions, such as expression of eNOS, and grow rapidly in culture without requirement for special growth factors, and thus are very often used as an *in vitro* model for endothelial cells [22], [23], [24], [25]. HUVECs and EA. hy926 cells were starved in DMEM containing 0.5% FBS for 24 hrs and then treated with glucose (25 mM) or MG (30 or 100 µM) in DMEM containing 10% FBS for 3 days.

### Small Interfering RNA (siRNA)

Knockdown of aldolase A or aldolase B was established by 24-h transfection of cells with a siRNA pool (a mixture of 3 or 4 different siRNA duplexes) targeting aldolase A or B (Santa Cruz Biotechnology Inc., Santa Cruz, CA, USA) in DharmaFECT™ 4 Transfection Reagent (Thermo Fisher, Nepean, ON, Canada). The non-targeting control siRNA pool is purchased from Santa Cruz (CA, USA). Briefly, transfection complexes were formed by incubating 100 µL siRNA pool (10 µM) with 25 µL of DharmaFECT™ 4 Transfection Reagent in 1 mL of serum-free DMEM for 20 min at room temperature. Transfection complexes were mixed with 4 mL of serum-free DMEM and added to cells. After 6 h of incubation, 5 mL DMEM supplemented with 20% FBS was added for a final siRNA concentration at 100 nM. After another 18 h, the transfection medium was replaced by 10% FBS DMEM with or without MG or high glucose and incubated for 3 days. Aldolase B mRNA was determined by a real-time PCR assay using SYBR Green PCR Master Mix (Bio-Rad) with the primers for human aldolase B (forward 5′-AGCCTCGCTATCCAGGAAAACG-3′, reverse 5′-TGGCAGTGTTCCAGGTCATGGT-3′) and β-actin (forward 5′-ACTTAGTTGCGTTACACCCTT-3′, reverse 5′-GTCACCTTCACCGTTCCA-3′). Primers for human aldolase A (Catalog Number QT00082460) and aldose reductase (Catalog Number QT01668695) were purchased from Qiagen (Mississauga, ON, Canada). The mRNA expression of aldolase A, aldolase B or aldose reductase was normalized relative to reference gene β-actin using ΔCt calculations [26].

**Figure 1:**
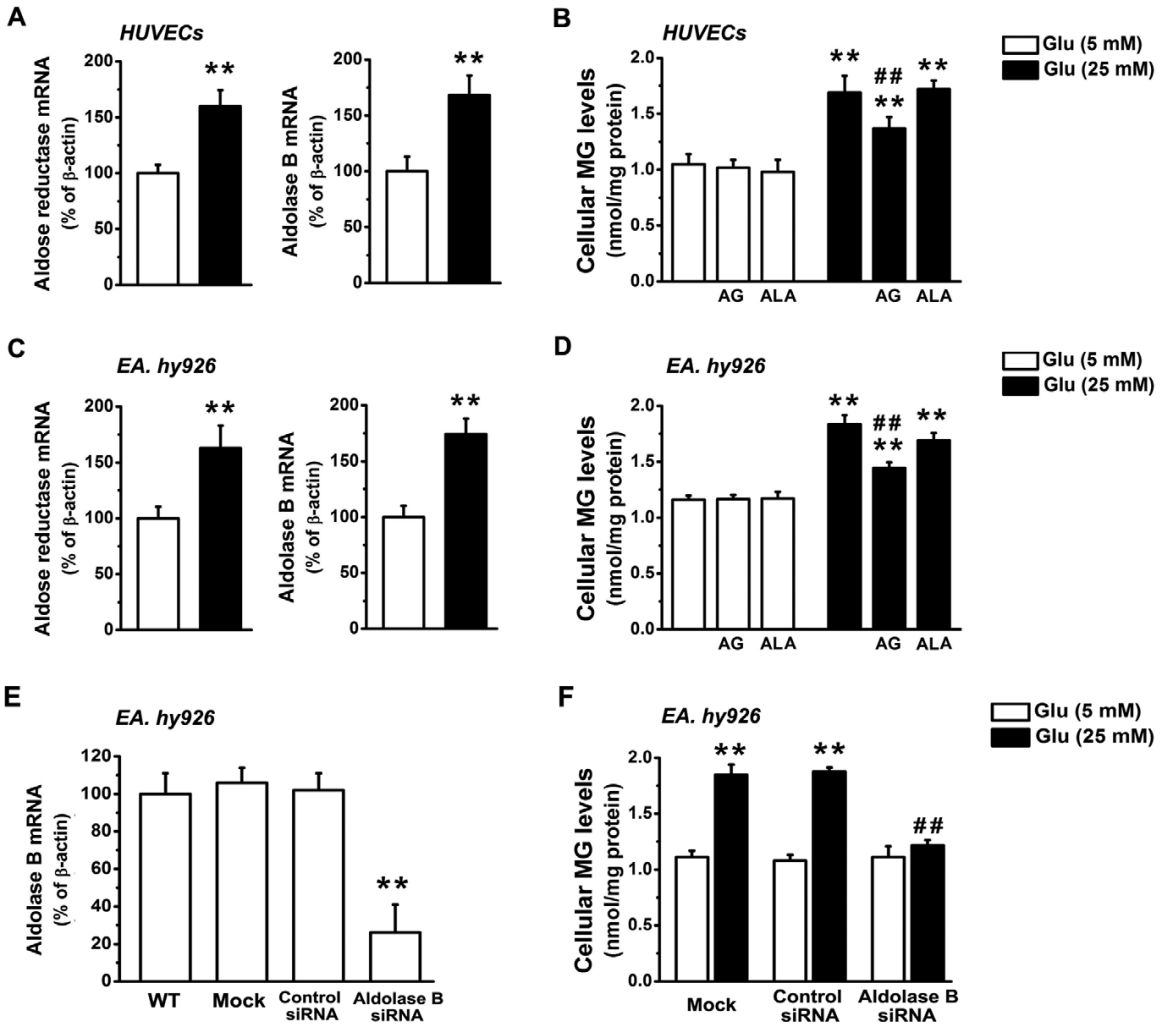
Knockdown of aldolase B prevented MG overproduction in high glucose-treated EA. hy926 cells. Real-time PCR analysis of aldose reductase and aldolase B expression (A, C) and MG levels (B, D) in human umbilical vein endothelial cells (HUVECs) (A, B) and EA. hy926 cells (C, D) treated with glucose (Glu) in the presence or absence of aminoguanidine (AG, 1 mM) and alagebrium (ALA, 100 µM) for 3 days. **P<0.01 *vs*. 5 mM glucose;^ ##^ *P*<0.01 *vs*. 25 mM glucose. Levels of aldolase B mRNA (E) and levels of MG (F) in wide-type cells (WT) and cells transfected with control or aldolase B siRNA, or only transfection agent (Mock). **P<0.01 *vs*. 5 mM glucose;^ ##^ *P*<0.01 *vs*. control siRNA.

### MG Measurement

MG levels were determined using our recently modified method [27]. Briefly, cells were sonicated three times for 5 seconds each time and centrifuged at 12,000 rpm (10 min, 4°C). The supernatant of 180 µL was incubated with 180 µL of perchloric acid (PCA, 1 N) and 40 µL of o-phenylenediamine (*o*-PD, 100 mM) for 24 h at room temperature in the dark. The mixture was centrifuged at 12,000 rpm (10 min, 4°C). The supernatant of 180 µL was mixed with 20 µL of 5-methylquinoxaline (5-MQ, internal standard) and analyzed by high-performance liquid chromatography (HPLC) with mobile phase buffer containing 17% acetonitrile, 8% 50 mM NaH_2_PO_4_ (pH 4.5), and 75% water.

### Confocal Imaging of AGEs

N(ε)-carboxyethyl-lysine (CEL) is a MG specific AGE formed by the reaction between MG and lysine residues in proteins [28]. CEL formation in EA. hy926 cells was visualized by confocal microscopy after immunofluorescent staining. Cells were cultured on glass coverslips, fixed and permeabilized with pre-cold methanol (20 min, −20°C) and blocked with goat serum in phosphate-buffered saline (PBS) (1∶30, 30 min), and then, incubated with mouse monoclonal CEL antibody (Cosmo bio, diluted 1∶250 in blocking solution, 3 h at room temperature or overnight at 4°C). Subsequently, the processed cell preparations were washed with PBS, and incubated with Alexa 488-conjugated secondary antibodies (Invitrogen, Burlington, ON, Canada, diluted 1∶300 in blocking solution, 2 h at room temperature). Finally, the prepared cells were washed again and mounted in mounting media with propidium iodide (Invitrogen, Burlington, ON, Canada). Thereafter, the slides were examined under a confocal microscope with the appropriate filters. The fluorescence intensity was determined using Image J by analyzing at least 50 random cells per sample.

### Measurement of Oxidative Stress

After different treatments, cells were washed with PBS containing 0.9 mM calcium chloride and 0.5 mM magnesium chloride, and then stained with a non-specific ROS probe (DCF–DA, Invitrogen, Burlington, ON, Canada) or a specific cell-permeable fluorogenic H_2_O_2_ probe (Calbiochem, San Diego, CA, USA). The H_2_O_2_ probe is a monosulfonated non-fluorescent fluorescein ester compound which selectively and sensitively reacts with H_2_O_2_ through a non-oxidative mechanism to yield corresponding fluorescein [29]. The fluorescence intensities of these probes were analyzed with a Fluoroskan Ascent plate reader (Thermo LabSystem, Franklin, MA, USA) as previously described [29], [30]. Protein oxidation was assessed by measuring total protein carbonyls with an immunoblot kit (Cell Biolabs Inc., San Diego, CA, USA). DNA oxidation biomarker 8-oxo-dG was visualized by immunofluorescent staining and photographed under a fluorescent microscopy, using a specific mouse monoclonal 8-oxo-dG antibody (Trevigen, Gaithersburg, MD, USA), following the manufacturer’s instructions.

### Western Blot Analysis

Cells were harvested and lysed in RIPA buffer (Santa Cruz Biotechnology Inc., Santa Cruz, CA, USA) supplemented with protease inhibitors. Total cellular proteins were fractionated by 10% SDS-PAGE and immunoblotted with antibodies as follows: *O*-GlcNAc (RL2) (1∶1000, Thermo Fisher, Nepean, ON, Canada), and α-tubulin (1∶500, Santa Cruz Biotechnology Inc., Santa Cruz, CA, USA). Nuclear proteins were extracted as previously described [31] and level of nuclear NF-κB was measured with antibodies against NF-κB (p65) (1∶500) and lamin B (1∶1000) purchased from Santa Cruz Biotechnology Inc., Santa Cruz, CA, USA.

### Membrane PKC Activity

Cells cultured on 100-mm dish were washed, scrapped off, suspended in 1 mL of Tris-sucrose buffer (20 mM Tris-base, 2 mM EDTA, 0.5 mM EGTA and 0.3 M sucrose, pH 7.4), and then added with protease inhibitors and homogenized by being passed 15 times through a 27.5 gauge needle. After centrifugation at 2,500 g (10 min, 4°C) to remove nuclei and cell debris, cell membrane was fractionated by a high speed ultra-centrifugation at 105,000 g (30 min, 4°C). Membrane pellets were washed with Tris buffer (20 mM Tris-base, 2 mM EDTA and 0.5 mM EGTA, pH 7.4), ultra-centrifuged (105,000 g, 30 min, 4°C) and re-suspended in 0.3 mL of Tris buffer with 0.5% Triton X-100 on ice for 1 h. The supernatant after ultra-centrifugation (105,000 g, 30 min, 4°C) was collected and membrane PKC proteins were purified through a DEAE cellulose (DE-52) column previously equilibrated with the Tris buffer. After washing the column with 3 mL of Tris buffer, the bound PKC was eluted with 0.5 ml Tris buffer containing 0.2 M NaCl. Membrane PKC activity was assessed by a PKC activity assay kit according to the manufacturer’s instructions (Assay Designs, Ann Arbor, MI, USA).

**Figure 2:**
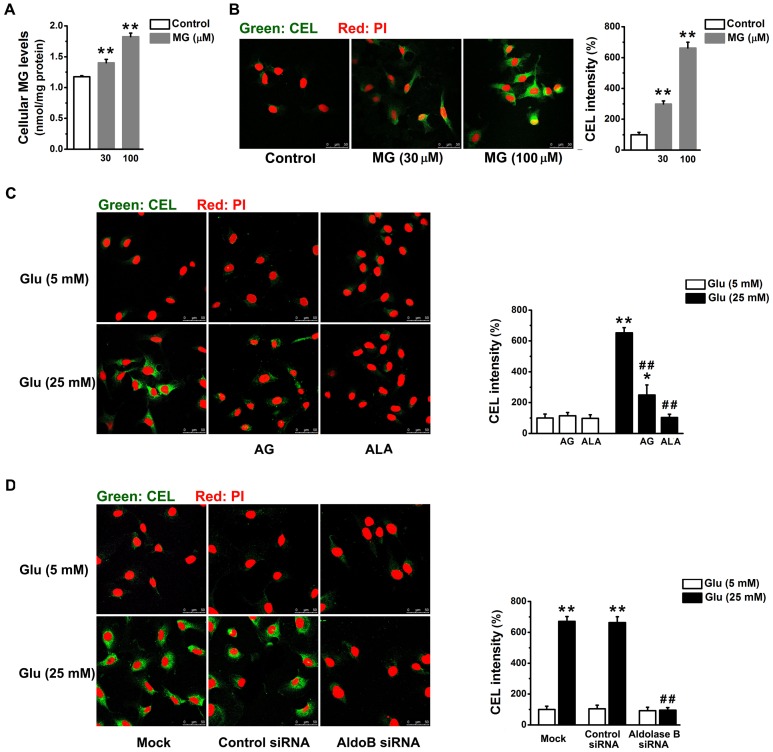
Knockdown of aldolase B prevented AGEs overproduction in high glucose-treated EA. hy926 cells. Levels of MG (A) and N(ε)-carboxyethyl-lysine (CEL) in green (B) in cells treated with exogenous MG for 3 days. **P<0.01 *vs*. control (5 mM glucose). (C) CEL levels in cells treated with glucose (Glu) in the presence or absence of aminoguanidine (AG, 1 mM) and alagebrium (ALA, 100 µM) for 3 days. *P<0.05, **P<0.01 *vs*. 5 mM glucose;^ ##^ *P*<0.01 *vs*. 25 mM glucose. (D) CEL levels in cells transfected with control or aldolase B siRNA, or only transfection agent (mock). **P<0.01 *vs*. 5 mM glucose; ^##^ *P*<0.01 *vs*. control siRNA. Nuclear DNA (red) was stained with propidium iodide (PI). The summary of fluorescence intensity of CEL was measured using Image J.

### Materials

(E)-2-(4-fluorophenethyl)-3-fluoroallylamine (MDL-72974) was a generous gift from Dr. Peter Yu (Department of Pharmacology, University of Saskatchewan, Canada). Alagebrium was a generous gift from Synvista Therapeutics (Montvale, NJ). Diallyl disulfide (DADS) and aminoguanidine was purchased from Sigma-Aldrich, Oakville, ON, Canada.

### Statistics

Data are expressed as mean ± SEM from at least five independent experiments (*n*≥5 in each group). Statistical analyses were performed using parametric Student’s t-test (two-tailed) or one-way ANOVA.

**Figure 3:**
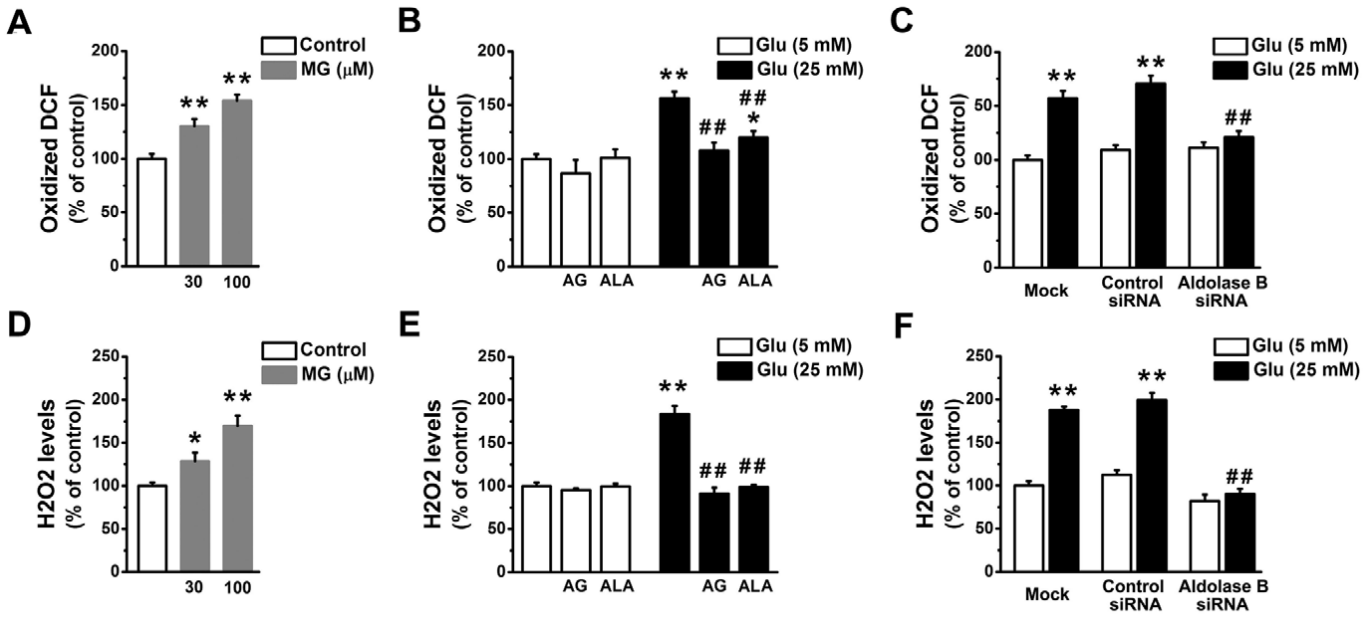
Knockdown of aldolase B prevented the increase of oxidized DCF and H_2_O_2_ levels in high glucose-treated EA. hy926 cells. Levels of oxidized DCF (A) in cells treated with exogenous MG (**P<0.01 vs. control), (B) in cells treated with glucose in the presence or absence of aminoguanidine (AG, 1 mM) and alagebrium (ALA, 100 µM) (*P<0.05, **P<0.01 vs. 5 mM glucose;^ ##^ *P*<0.01 vs. 25 mM glucose), and (C) in cells transfected with control or aldolase B siRNA, or only transfection agent (mock) (*P<0.05, **P<0.01 vs. 5 mM glucose; ^##^ *P*<0.01 vs. control siRNA). Levels of H_2_O_2_ (D) in cells treated with exogenous MG (*P<0.05, **P<0.01 vs. control), (E) in cells treated with glucose in the presence or absence of aminoguanidine (AG, 1 mM) and alagebrium (ALA, 100 µM) (**P<0.01 vs. 5 mM glucose;^ ##^ *P*<0.01 vs. 25 mM glucose), and (F) in cells transfected with control or aldolase B siRNA, or only transfection agent (mock) (**P<0.01 vs. 5 mM glucose; ^##^ *P*<0.01 vs. control siRNA).

## Results

### High Glucose Up-regulated Aldolase B and Increased MG Formation in HUVECs and EA. hy926 cells

High glucose (25 mM) treatment up-regulated aldose reductase (*P*<0.01) and aldolase B (*P*<0.01) mRNA expression and accelerated MG formation (*P*<0.01) in HUVECs (Figs. 1A, 1B). Cellular MG overproduction induced by high glucose was partially reduced by aminoguanidine (*P*<0.01), and not reduced by alagebrium (*P* = 0.78) (Fig. 1B).

To investigate the role of aldolase B in the high glucose-induced MG overproduction and endothelial cell dysfunction, HUVEC-derived EA. hy926 cells were used in this study. Similarly, high glucose up-regulated aldose reductase (*P*<0.01) and aldolase B (*P*<0.01) in EA. hy926 cells (Fig. 1C). High glucose increased MG production (*P*<0.01) in EA. hy926 cells, which was partially reduced by aminoguanidine (*P*<0.01) and unaltered by alagebrium (*P* = 0.19) (Fig. 1D).

### Knockdown of Aldolase B Prevented High Glucose-increased MG Formation in EA. hy926 cells

Transfection with aldolase B siRNA reduced cellular mRNA levels of aldolase B by 74%, and completely prevented high glucose-elevated formation of MG in EA. hy926 cells (Figs. 1E, 1F). Aldolase A, cytochrome P450 2E1 (CYP 2E1) and semicarbazide-sensitive amine oxidase (SSAO) are responsible for MG generation in glycolysis and in the metabolism of fatty acids and proteins, respectively [6]. However, high glucose-increased MG production in EA. hy926 cells was not affected by application of DADS (an inhibitor of CYP 2E1, 100 µM, *P* = 0.51) or MDL-72974 (an inhibitor of SSAO, 5 µM, *P* = 0.84), and by transfection with aldolase A siRNA (*P* = 0.24) which reduced cellular mRNA levels of aldolase A by 70% (data not shown).

### Knockdown of Aldolase B Prevented High Glucose-increased AGEs Formation in EA. hy926 cells

Since incubation of EA. hy926 cells with MG (100 µM) or glucose (25 mM) induced a similar increase in cellular MG levels (*P* = 0.88) (Fig. 2A), the direct effects of MG (30 or 100 µM) on endothelial cells were investigated. MG (30 or 100 µM, 3 days) elevated CEL levels (*P*<0.01) in EA. hy926 cells (Fig. 2B). Glucose (25 mM) treatment increased cellular CEL levels (*P*<0.01), which was similar with that induced by 100 µM MG (*P* = 0.71) (Figs. 2B, 2C). High glucose-induced CEL overproduction in endothelial cells was partially reduced by aminoguanidine (*P*<0.01) and completely prevented by alagebrium (*P*<0.01) or knockdown of aldolase B expression (*P*<0.01) (Figs. 2C, 2D).

### Knockdown of Aldolase B Prevented High Glucose-induced ROS in EA. hy926 cells

High glucose-increased production of ROS is regarded as an important contributor to endothelial dysfunction in diabetic vascular complications [32]. MG (30 or 100 µM, 3 days) elevated levels of oxidized DCF (a marker of total cellular ROS) in EA. hy926 cells (*P*<0.01) (Fig. 3A). The increase in oxidized DCF levels induced by MG (100 µM) was similar with that induced by glucose (25 mM) (*P* = 0.79) (Figs. 3A, 3B). High glucose-increased cellular formation of oxidized DCF was totally abolished by aminoguanidine (*P*<0.01) or by transfection with aldolase B siRNA (*P*<0.01), and partially reduced by alagebrium (*P*<0.01) (Figs. 3B, 3C). Cellular levels of H_2_O_2_ were similarly elevated by MG (100 µM) and glucose (25 mM) (*P* = 0.54) (Figs. 3D, 3E). Application of aminoguanidine or alagebrium, or transfection of aldolase B siRNA prevented the formation of H_2_O_2_ (*P*<0.01) in high glucose-treated endothelial cells (Figs. 3E, 3F).

### Knockdown of Aldolase B Prevented High Glucose-induced Protein and DNA Oxidation in EA. hy926 cells

**Figure 4:**
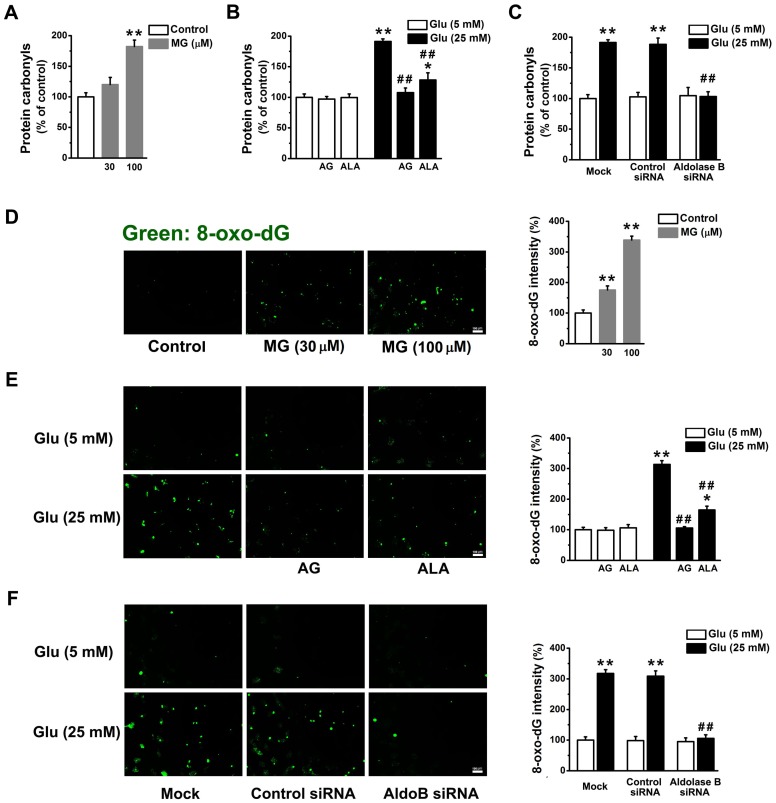
Knockdown of aldolase B prevented protein and DNA oxidation in high glucose-treated EA. hy926 cells. Levels of protein carbonyls (A) in cells treated with exogenous MG (**P<0.01 vs. control), (B) in cells treated with glucose in the presence or absence of aminoguanidine (AG, 1 mM) and alagebrium (ALA, 100 µM) (*P<0.05, **P<0.01 vs. 5 mM glucose;^ ##^ *P*<0.01 vs. 25 mM glucose), and (C) in cells transfected with control or aldolase B siRNA, or only transfection agent (mock) (**P<0.01 vs. 5 mM glucose; ^##^ *P*<0.01 vs. control siRNA). Levels of 8-oxo-dG (D) in cells treated with exogenous MG (**P<0.01 vs. control), (E) in cells treated with glucose in the presence or absence of aminoguanidine (AG, 1 mM) and alagebrium (ALA, 100 µM) (*P<0.05, **P<0.01 vs. 5 mM glucose;^ ##^ *P*<0.01 vs. 25 mM glucose), and (F) in cells transfected with control or aldolase B siRNA, or only transfection agent (mock) (**P<0.01 vs. 5 mM glucose; ^##^ *P*<0.01 vs. control siRNA). Fluorescence intensity of 8-oxo-dG per cell was measured using Image J.

MG (100 µM) and glucose (25 mM) induced similar increases in levels of protein carbonyls (a marker of protein oxidation) in EA. hy926 cells (*P* = 0.85) (Figs. 4A, 4B). Application of aminoguanidine or transfection of aldolase B siRNA totally abolished high glucose-increased levels of protein carbonyls (*P*<0.01). Application of alagebrium partially reduced the formation of protein carbonyls in high glucose-treated cells (*P*<0.01) (Figs. 4B, 4C).

MG (30 or 100 µM, 3 days) elevated levels of 8-oxo-dG (a marker of DNA oxidation) in EA. hy926 cells (*P*<0.01) (Fig. 4D). The increase of 8-oxo-dG levels induced by MG (100 µM) was similar to that induced by 25 mM glucose (*P* = 0.26) (Figs. 4D, 4E). High glucose-increased 8-oxo-dG levels were prevented by aminoguanidine or by transfection with aldolase B siRNA, and partially reduced by alagebrium (*P*<0.01) (Figs. 4E, 4F).

### Knockdown of Aldolase B Blocked High Glucose-activated Metabolic or Signalling Pathways in EA. hy926 cells

**Figure 5:**
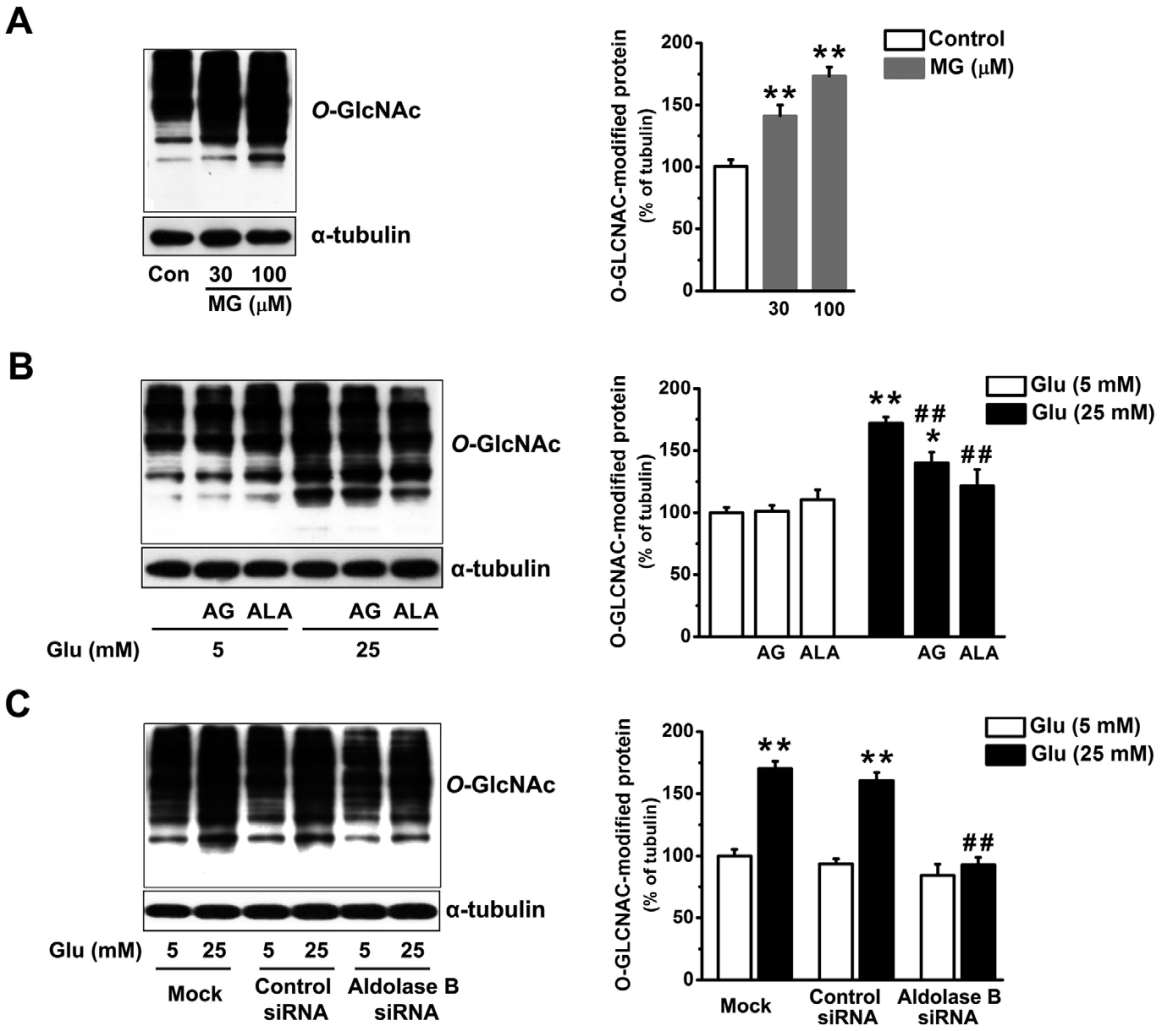
Knockdown of aldolase B prevented high glucose-increased O-linked N-acetyl glucosamine (*O*-GlcNAc) modification in EA. hy926 cells. (A) *O*-GlcNAc modification of total cellular proteins in cells treated with exogenous MG for 3 days. **P<0.01 *vs*. control (5 mM glucose). (B) *O*-GlcNAc modification of total cellular proteins in cells treated with glucose (Glu) in the presence or absence of aminoguanidine (AG, 1 mM) and alagebrium (ALA, 100 µM). *P<0.05, **P<0.01 *vs*. 5 mM glucose;^ ##^ *P*<0.01 vs. 25 mM glucose. (C) *O*-GlcNAc modification of total cellular proteins in cells transfected with control or aldolase B siRNA, or only transfection agent (mock). **P<0.01 *vs*. 5 mM glucose; ^##^ *P*<0.01 *vs*. control siRNA.

Activation of the hexosamine pathway by high glucose causes an elevated *O*-GlcNAc modification of nuclear and cytosolic proteins [1]. MG (30 or 100 µM) increased *O*-GlcNAc modification of proteins in EA. hy926 cells (*P*<0.01) (Fig. 5A). Glucose (25 mM)-elevated *O*-GlcNAc modification was similar to that induced by MG (100 µM) (*P* = 0.76), and totally abolished by alagebrium (*P*<0.01) or by transfection with aldolase B siRNA (*P*<0.01) but only partially reversed by aminoguanidine (*P*<0.01) (Figs. 5B, 5C).

**Figure 6:**
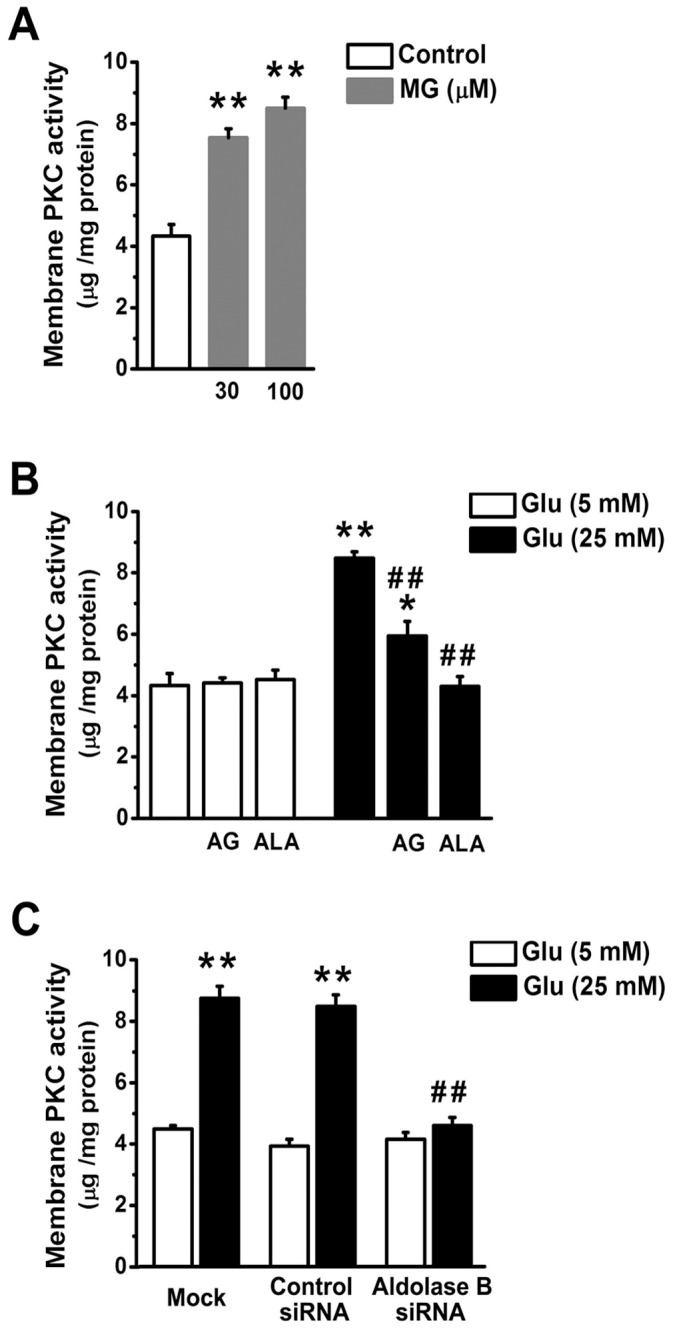
Knockdown of aldolase B prevented high glucose-increased membrane protein kinase C (PKC) activities in EA. hy926 cells. (A) Membrane PKC activities in cells treated with exogenous MG for 3 days. **P<0.01 *vs*. control (5 mM glucose). (B) Membrane PKC activities in cells treated with glucose (Glu) in the presence or absence of aminoguanidine (AG, 1 mM) and alagebrium (ALA, 100 µM). *P<0.05, **P<0.01 *vs*. 5 mM glucose;^ ##^ *P*<0.01 *vs*. 25 mM glucose. (C) Membrane PKC activities in cells transfected with control or aldolase B siRNA, or only transfection agent (mock). **P<0.01 *vs*. 5 mM glucose; ^##^ *P*<0.01 *vs*. control siRNA.

Activation of PKC leads to its translocation to the plasma membrane where it catalyzes the phosphorylation of various substrates and mediates a diverse variety of biological processes [33]. MG (30 or 100 µM) elevated plasma membrane PKC activities in EA. hy926 cells (*P*<0.01) (Fig. 6A). High glucose (25 mM) incubation induced a similar elevation in the plasma membrane PKC activities as did 100 µM MG (*P* = 0.69) (Figs. 6A, 6B). High glucose-elevated plasma membrane PKC activity was prevented by alagebrium (*P*<0.01) or by knockdown of aldolase B (*P*<0.01), but only partially by aminoguanidine (*P*<0.01) (Figs. 6B, 6C).

**Figure 7:**
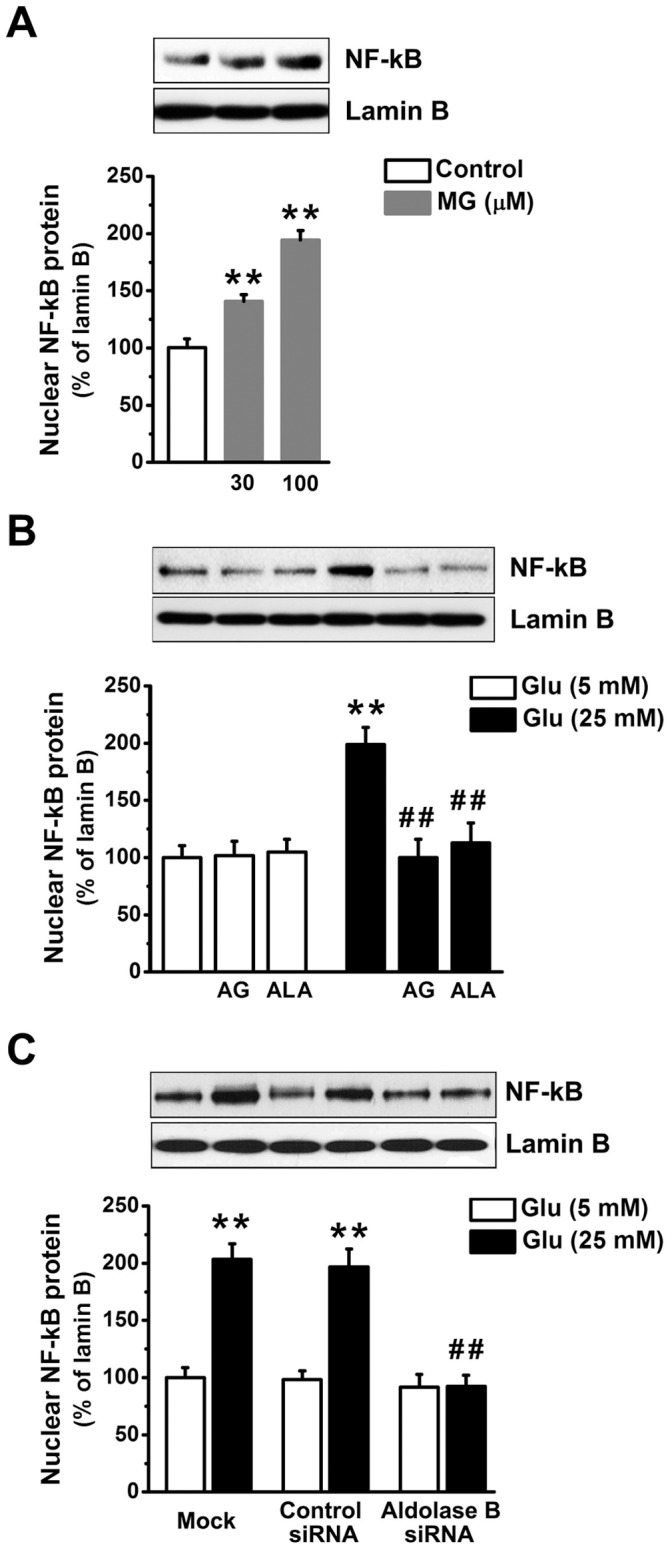
Knockdown of aldolase B prevented high glucose-increased NF-κB nuclear translocation in EA. hy926 cells. (A) Nuclear p65 subunit of NF-κB in cells treated with exogenous MG for 3 days. **P<0.01 *vs*. control (5 mM glucose). (B) Nuclear p65 subunit of NF-κB in cells treated with glucose (Glu) in the presence or absence of aminoguanidine (AG, 1 mM) and alagebrium (ALA, 100 µM). **P<0.01 *vs*. 5 mM glucose;^ ##^ *P*<0.01 *vs*. 25 mM glucose. (C) Nuclear p65 subunit of NF-κB in cells transfected with control or aldolase B siRNA, or only transfection agent (mock). **P<0.01 *vs*. 5 mM glucose; ^##^ *P*<0.01 *vs*. control siRNA.

Activated NF-κB (p50/p65 dimer) translocates into the nucleus and regulates the expression of a large number of genes involved in immune and inflammatory response, apoptosis, cell proliferation and differentiation [34]. MG (30 or 100 µM) increased nuclear p65 subunit of NF-κB in EA. hy926 cells (*P*<0.01) (Fig. 7A). High glucose-elevated nuclear amount of NF-κB p65 was similar with that induced by 100 µM MG (*P* = 0.82), and prevented by alagebrium (*P*<0.01), aminoguanidine (*P*<0.01), and aldolase B knockdown (*P*<0.01) (Figs. 7A, 7B, 7C).

## Discussion

Our present work demonstrated that siRNA knockdown of aldolase B blocked high glucose-activated metabolic and signaling pathways by the normalization of MG production in endothelial cells. This revelation was substantiated by the following observations: 1) Treatment of EA. hy926 cells with MG (100 µM) and high glucose (25 mM) induced a similar increase in cellular MG levels and a similar activation of biochemical pathways involved in hyperglycemic damage; 2) Aldolase B is the major enzyme for high glucose-increased MG production in endothelial cells because aldolase B knockdown completely inhibited MG overproduction in high glucose-treated EA. hy926 cells; 3) Both knockdown of aldolase B and the application of aminoguanidine or alagebrium prevented high glucose-activated metabolic and signaling pathways in EA. hy926 cells.

Enhanced accumulation of MG is postulated to be one of the important molecular mechanisms leading to endothelial dysfunction and diabetic vascular complications [1], [5], [8]. We previously reported that incubation with MG (30 or 100 µM) and glucose (25 mM) similarly decreased NO synthase activity and NO production in cultured endothelial cells and reduced endothelium-dependent muscle relaxation in rat aorta [8]. Our present study further validates the role of MG as an upstream activator for hyperglycemia-induced metabolic and signaling changes. MG is the major precursor of AGEs in endothelial cells [10]. MG treatment elevated AGEs (as estimated by intracellular CEL) levels in EA. hy926 cells (Fig. 2). AGEs alter protein structures and functions. For example, MG-modified extracellular matrix molecules impaired matrix-matrix interactions and increased the stiffness of the vasculature [35]. In addition, AGEs can activate their specific receptors on endothelial cells and cause cellular perturbation, such as increased permeability, oxidative stress, activation of NF-κB and vascular inflammation [35], [36]. MG is also a pro-oxidant [30], [37]. A possible reason is a MG-induced decrease in mitochondrial complex III and SOD activities [38]. Here we found MG enhanced the formation of ROS and oxidation of protein and DNA in EA. hy926 cells (Figs. 3, 4). Moreover, we provided the first evidence that treatment with MG can directly stimulate *O*-GlcNAc modification and plasma membrane PKC activation (Figs. 5, 6). It is well known that abnormal activation of PKC by high glucose decreased NO production and increased vasoconstrictor endothelin-1 (ET-1) and ROS production in endothelial cells [1]. Increased *O*-GlcNAc modification of proteins appears to be important in the pathogenesis of endothelial dysfunction. For example, high glucose-increased *O*-GlcNAc modification of endothelial NO synthase decreased its activity in endothelial cells [39]. We also found that MG directly activated NF-κB by stimulating its nuclear translocation in endothelial cells (Fig. 7). High glucose-activated NF-κB via AGEs, ROS or PKC increased expression of genes contributing to endothelial dysfunction, such as ET-1, adhesion molecules and inflammatory cytokines [35], [40], [41].

Aldolase B is a major enzyme responsible for high glucose-induced MG overproduction in VSMCs and the aorta [6]. Triosephosphates glyceraldehyde 3-phosphate (GA3P) and dihydroxyacetone phosphate (DHAP) are considered major sources for endogenous MG formation and showed high efficiencies of non-enzymatic conversion to MG [6], [42], [43]. In cells, fructose is quickly phosphorylated to fructose 1-phosphate (F-1-P), which is cleaved by aldolase B to generate GA3P and DHAP [44]. Fructose is produced from glucose via the polyol pathway [1], [6], [45]. On the other hand, glucose is metabolized enzymatically through the glycolytic pathway into fructose-1,6-diphosphate, which subsequently forms GA3P and DHAP catalyzed by aldolase A [44]. Secondary sources of MG include the oxidation of aminoacetone by SSAO and the oxidation of acetone by CYP 2E1 [46]. We have recently reported that aldolase B, but not aldolase A, SSAO or CYP 2E1, was up-regulated and MG was over-produced in the aorta of diabetic rats; knockdown of aldolase B prevented high glucose-elevated MG formation in VSMCs [6]. In the present work, we observed that high glucose up-regulated aldose reductase (the first and rate-limiting enzyme of the polyol pathway [45]) and aldolase B gene expression and increased MG formation in endothelial cells (Fig. 1). siRNA knockdown of aldolase B completely inhibited the excess MG generation in glucose-treated endothelial cells (Fig. 1). However, siRNA knockdown of aldolase A or inhibition of SSAO or CYP 2E1 had no effect on glucose-increased cellular MG overproduction (data not shown). These data indicate that aldolase B is predominantly responsible for glucose-increased MG formation in endothelial cells and the inhibition of MG formation is solely responsible for the observed effects of aldolase B knockdown on high glucose-activated metabolic and signaling pathways.

Aminoguanidine and alagebrium are the most widely used MG scavenger and AGEs breaker, respectively [21]. The inhibitory effects of aminoguanidine or alagebrium on high glucose-induced endothelial abnormalities confirm the role of MG as a mediator for high glucose-activated cellular pathways (Figs. 2, 3, 4, 5, 6, 7). However, our work also reveals limitations of aminoguanidine or alagebrium in accurately evaluating MG’s contribution to endothelial dysfunction and diabetic complications, when compared with the knockdown of aldolase B which specifically prevents glucose-induced MG overproduction. Aminoguanidine is a non-specific MG scavenger. Its guanidine residue can react with the carbonyl in MG or in other carbonyl compounds, such as 3-deoxyglucosone and malondialdehyde [21]. Moreover, aminoguanidine can react directly with ROS, such as H_2_O_2_, hydroxyl radical and peroxynitrite [47]. We found that the application of aminoguanidine completely abolished oxidative stress, but only partially decreased MG and CEL production, *O*-GlcNAc modification and plasma membrane PKC activities in high glucose-treated cells (Figs. 1, 2, 3, 4, 5, 6). Alagebrium breaks the established AGE crosslinks [21]. The application of alagebrium completely inhibited the formation of CEL, but it did not change MG levels in high glucose-treated EA. hy926 cells (Figs. 1, 2). In addition to the formation of AGEs, MG also stimulates the formation of ROS [38]. Our studies showed that alagebrium only partially reduced the formation of oxidized DCF, protein carbonyls and 8-oxo-dG in high glucose-treated EA. hy926 cells (Figs. 3, 4), although it can react with H_2_O_2_ in the test tube [48] and completely reduced the glucose-increased H_2_O_2_ formation in our test cells (Fig. 3).

In conclusion, MG directly mediates high glucose-induced production of AGEs, oxidative stress, and increases in *O*-GlcNAc modification and protein levels or activities of protein kinase C and NF-κB in endothelial cells. More importantly, this study demonstrates that aldolase B is the major enzyme for high glucose-increased MG production in endothelial cells. Knockdown of aldolase B prevents MG overproduction and, by doing so, blocks high glucose-induced activation of multiple metabolic and signaling pathways in endothelial cells.
